# Supplementary material for: Disease Manifestations in Mucopolysaccharidoses and Their Impact on Anaesthesia-Related Complications—A Retrospective Analysis of 99 Patients
Source: J Clin Med. 2021 Aug 10;10(16):3518. doi: 10.3390/jcm10163518 (PMC8397084; doi:10.3390/jcm10163518)
Supplement: Supplementary file 1 [file jcm-10-03518-s001.zip › jcm-1312181-supplementary.pdf]

Table S1. Cervical spine disease in MPS.

	Stenosis	Stability		
		Stable	Instable	Surgical Fusion
**MPSIH**	Normal	10	1	-
**N = 32**	Stenosis	6	6	-
	Stenosis with myelopathy	1	2	-
	Decompression surgery	1	2	3
**MPSIS**	Normal	1	-	-
**N = 3**	Stenosis	-	-	-
	Stenosis with myelopathy	1	-	-
	Decompression surgery	1	-	-
**MPSII**	Normal	8	3	-
**N = 16**	Stenosis	3	-	-
	Stenosis with myelopathy	2	-	-
	Decompression surgery	-	-	-
**MPSIII**	Normal	33	-	-
**N = 38**	Stenosis	4	-	-
	Stenosis with myelopathy	-	-	-
	Decompression surgery	-	-	-
**MPSIV**	Normal	1	-	-
**N = 6**	Stenosis	1	3	-
	Stenosis with myelopathy	-	-	-
	Decompression surgery	1	-	1
**MPSVI**	Normal	-	-	-
**N = 4**	Stenosis	1	1	-
	Stenosis with myelopathy	-	-	-
	Decompression surgery	-	1	1

Table S2. Characteristics or anaesthetic cases.

Characteristics	Overall N = 484	MPSIH N = 224	MPSIS N = 20	MPSII N = 62	MPSIII N = 126	MPSIV N = 29	MPSVI N = 23
**Cases**							
Age (yrs)[1]	6.1 (0.7-38.7)	5.2 (0.8-23.8)	21.6 (7.1-29.1)	8.3 (0.7-32.1)	5.3 (1.1-38.7)	10.3 (4.1-18.6)	21.7 (7.5-38.2)
Weight (Z-Score)[2]	-0.6 (2.3)	-1.0 (1.9)	-1.8 (0.6)	0.6 (2.3)	0.7 (1.9)	-3.2 (1.5)	-4.9 (2.6)
Height (Z-Score)[2]	-1.9 (2.8)	-2.7 (2.5)	-4.1 (1.8)	-0.4 (2.6)	0.2 (1.9)	-4.2 (1.7)	-6.0 (1.4)
Present respiratory infection, n (%)	106 (22)	37 (17)	2 (10)	22 (35)	39 (31)	3 (10)	3 (13)
**Procedural information**							
Indication, n (%)							
Diagnostics only	106 (22)	52 (23)	-	15 (24)	35 (28)	4 (14)	-
Intervention	56 (12)	35 (16)	-	3 (4.8)	16 (13)	-	2 (8.7)
Minor surgery	235 (49)	99 (44)	17 (85)	27 (44)	60 (48)	18 (62)	14 (61)
Airway surgery	57 (12)	22 (9.8)	-	14 (23)	14 (11)	4 (14)	3 (13)
Major surgery	30 (6.2)	16 (7.1)	3 (15)	3 (4.8)	1 (0.8)	3 (10)	4 (17)
No. of procedures during anesthesia[1]	1.0 (1.0-5.0)	1.0 (1.0-4.0)	1.0 (1.0-2.0)	1.0 (1.0-4.0)	1.0 (1.0-5.0)	1.0 (1.0-5.0)	1.0 (1.0-2.0)
Emergency surgery, n (%)	28 (5.8)	11 (4.9)	6 (30)	4 (6.5)	5 (4.0)	-	2 (8.7)
Duration (h)[1]	2.1 (0.1-12.5)	2.0 (0.2-12.5)	3.4 (0.4-9.5)	2.5 (0.7-9.1)	2.3 (0.1-6.0)	2.3 (0.1-7.8)	1.4 (0.3-10.4)
Postoperative ICU care, n (%)	201 (54)	90 (54)	10 (83)	42 (70)	45 (40)	7 (64)	7 (88)
**Technical information**							
Type of aneasthesia, n (%)							
Standby	12 (2.5)	2 (0.9)	1 (5.0)	-	-	-	9 (39)
Sedation	85 (18)	33 (15)	-	7 (11)	40 (32)	3 (10)	2 (8.7)
Regional only	4 (0.8)	-	2 (10)	2 (3.2)	-	-	-
Balanced	95 (20)	46 (21)	2 (10)	19 (31)	18 (14)	5 (17)	5 (22)
TIVA	288 (60)	143 (64)	15 (75)	34 (55)	68 (54)	21 (72)	7 (30)
Primary airway approach, n (%)							
No airway	102 (22)	36 (16)	3 (15)	10 (18)	39 (31)	3 (10)	11 (50)
Laryngeal mask	97 (21)	66 (30)	1 (5.0)	6 (11)	13 (10)	8 (28)	3 (14)
Direct laryngoscopy	97 (21)	42 (19)	3 (15)	7 (12)	34 (27)	10 (34)	1 (4.5)
Videolaryngoscopy	42 (8.9)	13 (5.9)	2 (10)	2 (3.6)	22 (18)	3 (10)	-
Fibreoptic	46 (9.7)	19 (8.6)	10 (50)	13 (23)	1 (0.8)	-	3 (14)
FOI-SGA	78 (17)	39 (18)	-	17 (30)	16 (13)	5 (17)	1 (4.5)
Other	10 (2.1)	5 (2.3)	1 (5.0)	1 (1.8)	-	-	3 (14)
Difficult mask-ventilation, n (%)	14 (4.5)	5 (3.0)	3 (37.5)	5 (13.2)	1 (1.2)	-	-
Difficult larynx mask, n (%)	15 (8.1)	9 (8.0)	1 (33.3)	4 (14.8)	-	-	1 (25.0)
Difficult intubation, n (%)	55 (20.0)	34 (27.9)	7 (41.2)	10 (23.8)	1 (1.4)	-	3 (50.0)

[1] Median (Range)

[2] Mean (SD)

Abbreviations: FOI-SGA, fiberoptic intubation through a supraglottic airway; ICU, intensive care unit; TIVA, total intravenous anaesthesia.

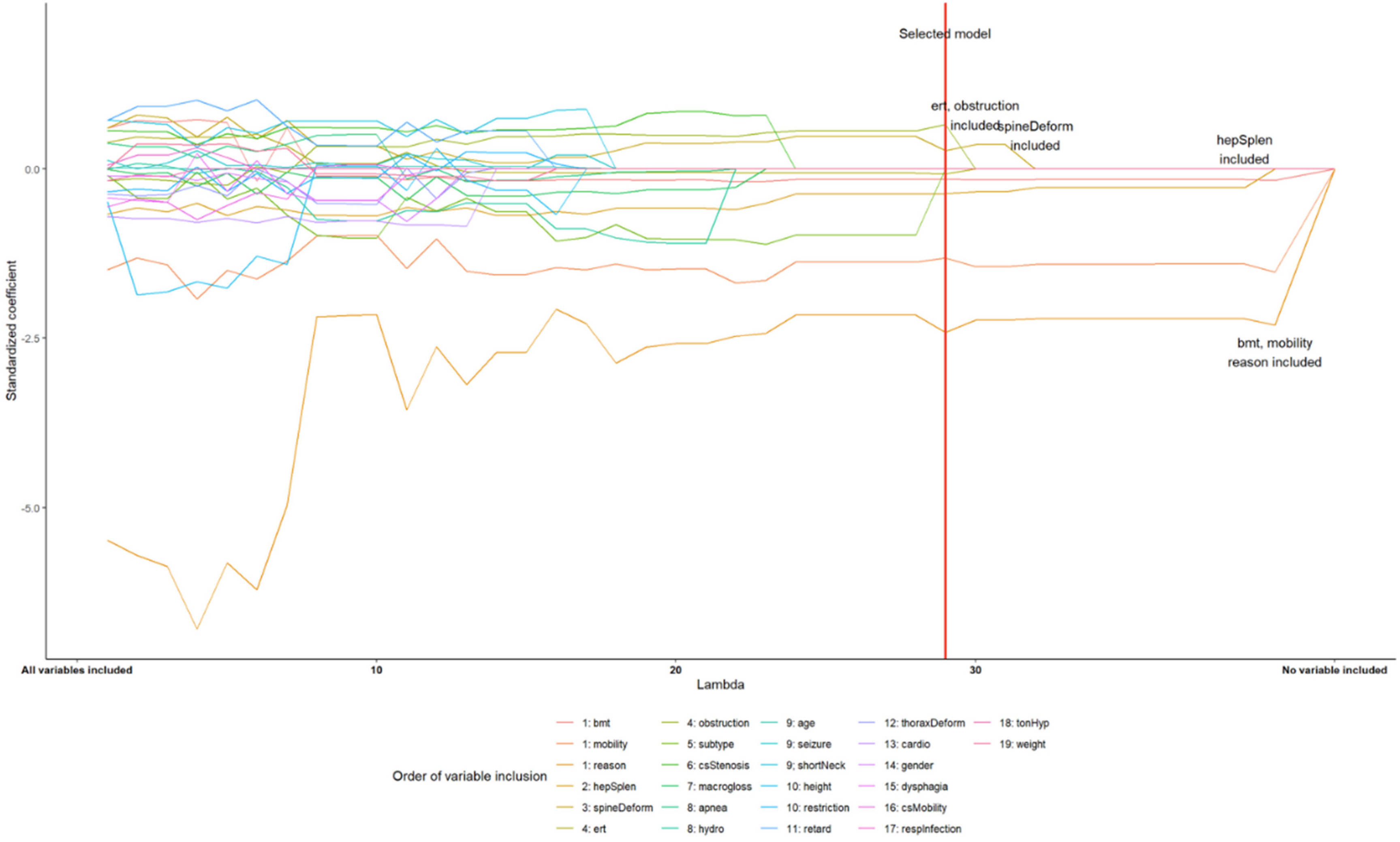

Figure S1. Variable selection path for the model based on the lasso method.
